# Sex Differences in Facial, Prosodic, and Social Context Emotional Recognition in Early-Onset Schizophrenia

**DOI:** 10.1155/2012/584725

**Published:** 2012-03-01

**Authors:** Julieta Ramos-Loyo, Leonor Mora-Reynoso, Luis Miguel Sánchez-Loyo, Virginia Medina-Hernández

**Affiliations:** ^1^Instituto de Neurociencias, CUCBA, Universidad de Guadalajara, 44130 Guadalajara, JAL, Mexico; ^2^Departamento de Neurociencias, CUCS, Universidad de Guadalajara, 44130 Guadalajara, JAL, Mexico; ^3^Centro Comunitario de Salud Mental No. 1, Guadalajara, 45150 Zapopan, JAL, Mexico

## Abstract

The purpose of the present study was to determine sex differences in facial, prosodic, and social context emotional recognition in schizophrenia (SCH). Thirty-eight patients (SCH, 20 females) and 38 healthy controls (CON, 20 females) participated in the study. Clinical scales (BPRS and PANSS) and an Affective States Scale were applied, as well as tasks to evaluate facial, prosodic, and within a social context emotional recognition. SCH showed lower accuracy and longer response times than CON, but no significant sex differences were observed in either facial or prosody recognition. In social context emotions, however, females showed higher empathy than males with respect to happiness in both groups. SCH reported being more identified with sad films than CON and females more with fear than males. The results of this study confirm the deficits of emotional recognition in male and female patients with schizophrenia compared to healthy subjects. Sex differences were detected in relation to social context emotions and facial and prosodic recognition depending on age.

## 1. Introduction

Sex differences in schizophrenia regarding to clinical, neuroanatomical, cognitive, emotional and social domains have been reported (i.e.,[1–6]). With respect to psychopathological characteristics, male patients suffer more acute symptoms than females, with higher prevalence of paranoid symptoms, aggression, and antisocial behavior [7]. The latter, on the other hand, show more affective disorders, such as anxiety and depression [8], as well as sudden changes in appetite, weight, and sexual activity [9]. Schizophrenic females also show a less deteriorative course during the illness [10], a better premorbid adjustment in the social, sexual, and marital domains, and improved outcomes with a higher index of spontaneous remissions and better treatment response than males, probably due to the protector effects of sexual hormones [11–13]. Other authors have pointed out that females are more capable of living independently, while male patients are more used to living in sheltered houses [13]. Furthermore, males present a higher number of hospitalizations and greater deterioration; thus, their outcomes and social reintegration tend to be unfavourable [7].

Some studies have reported sex differences in cognitive functions in schizophrenia [3, 14–16]. Results of these studies suggest that females perform better than males in executive functions, visual working memory, verbal memory, and learning. In this regard, schizophrenic females may be less vulnerable to cognitive deficits than their males counterparts although the former still show lower performance than healthy controls on different tasks [6, 17–20]. However, studies regarding sex differences in affect recognition in cases of schizophrenia are scarce. One showed that schizophrenic females outperform males when asked to label negative facial emotions, a skill that may contribute to improving their abilities in social life [21]. Weiss et al. [22] found that males with schizophrenia tend to interpret neutral faces as anger, while females interpret them as sadness. In addition, females outperform schizophrenic males in emotional prosodic and semantic processing [23]. In another study, an effect of sex was found in both facial and prosodic emotional recognition performance, where schizophrenic females were observed to be more accurate than men though they still presented difficulties in these processes when compared to control subjects [24].

Regarding sex differences in affect recognition abilities in healthy subjects, several studies have revealed that females generally outperform males, especially for negative facial emotional expressions [25–31]. Sex differences in emotional prosody have also been reported, showing that females can integrate both semantic content and emotional intonation information more quickly than males [32]. 

Results from various studies that included males exclusively or males and females together have demonstrated that schizophrenics show impairments in recognizing, expressing and regulating emotions [33–39]. Impairments in facial emotional recognition in schizophrenic patients are present from early stages of the illness and are greater for negative emotional stimuli, especially for fear and disgust [34, 40–42]. Difficulties in prosodic recognition have also been described in schizophrenia mainly for sadness and fear [23, 34, 36, 38, 43]. However, few studies have explored emotional recognition deficits in schizophrenic patients within social contexts. Studying impairments in emotional recognition in all modalities is relevant since this element contributes to abnormal social functioning, mainly in patients with a predominancy of negative symptoms [44, 45].

The question here is whether schizophrenic patients present similar sex differences for emotional recognition to those found in healthy subjects, based on an assessment of three modalities: facial, prosodic, and within a social context. Studying these three different modalities in the same subjects will make it possible to ascertain whether recognition disorders are similar in visual and prosodic recognition, as a general effect on emotion, and if they are present in a more ecological and complex modality, that is, a social context in which people express similar or different emotions in a dynamic behavioural pattern. It is possible than it in social contexts, the disorders observed in specific visual or auditory modalities could be compensated to recognize an expressed emotion, based on the conjunction of different sensory information. Furthermore, this approach will be able to evaluate other socioemotional aspects, such as the empathy level experienced by an observer while watching a film. Given the foregoing, one would expect that schizophrenic patients would show a poorer performance than healthy controls in emotional recognition tasks and, moreover, that females would show a greater preservation of emotional recognition than men.

Based on the findings reviewed above, the aim of the present study was to determine the existence of sex differences in facial, prosodic, and social context emotional recognition in patients with schizophrenia when compared to healthy controls.

## 2. Methods

### 2.1. Participants

Schizophrenic patients were recruited from the Guadalajara Mental Health Center of the Mexican Social Security Institute (IMSS). Thirty-eight paranoid schizophrenic patients (SCH 20 females and 18 males) with recent onset illness (from 6 months to 4 years) and 38 healthy control subjects (CON, 20 females and 18 males) matched with the patients for age (18–45 years) and years of formal education (minimum 9 years) met the inclusion criteria. Patients who had been submitted to electroconvulsive therapy within the previous 6 months, or had presented severe psychiatric comorbidity, were not included in the sample. All subjects were right-handed, according to Annette's test [46] but showed no neurological or chronic-degenerative disorders, addictions, carcinoma, diabetes, or infections. 

In addition, the female menstrual cycle was considered when programming the evaluations, such that they were distributed similarly across the different menstrual phases. This was done in order to avoid hormonal effects on emotional recognition, since some authors [47] have found that females perform more accurately in recognizing emotional expressions of fear during the preovulatory phase compared to the menstrual phase. Also, better recognition of emotions was found in the follicular phase, in association with stronger amygdala and orbitofrontal activation in females [48, 49].

 Patients were clinically assessed in two moments by an experienced psychiatrist, taking into account their clinical medical history and the international DSM-IV criteria [50]. Symptoms were rated by means of the Brief Psychiatric Rating Scale (BPRS, [51]) and the Positive and Negative Symptom Scale (PANSS, [52]).

The study was approved by the Ethic Committees of the IMSS and the Neuroscience Institute of the University of Guadalajara and was conducted according to Code of Ethics of the World Medical Association (Declaration of Helsinki). All subjects gave their informed consent before participating in the study.

### 2.2. Procedure

Patients arrived at the hospital in acute stage. In that moment, the first psychopathological assessment was conducted. The second psychopathological assesment was carried out during a remission stage under neuroleptic treatment. In this second session, the Affective States Scale was applied. This scale was also applied to control subjects. Subsequently, facial, prosodic, and social context emotional tasks were counterbalanced administered to the participants.

#### 2.2.1. Clinical Assessment

The first psychopathological assessment was conducted when patients arrived to the psychiatric hospital in acute condition without medication for their first admission due to diagnoses of schizophrenia. Thereafter, patients remained at the hospital for at least 15 days. The second assesment was conducted after approximately 15 days of hospitalization (females X = 14.25 ± 4.12, males X = 15.33 ± 5.58), once positive symptom were remitted under pharmacological treatment with typical (haloperidol: 15 mg/day) or atypical (olanzapine: 10 mg/day) neuroleptics. With the two completed assessments, it was possible to test whether there were clinical sex differences in both the acute phase and/or after a similar neuroleptic treatment with an equal dosage for all patients. A structured clinical interview was also applied to all subjects, as well as the laterality test [46] to confirm right handedness. These procedures were performed by one of the researchers, who is an experienced psychiatrist (VMH). Only the BPRS and PANSS were applied in both sessions.

#### 2.2.2. Affective States Scale

The Affective States Scale was applied to controls and patients, in order to determine their basal emotional state, as this could influence their performance on the emotional recognition tasks. At the beginning of the evaluation session, participants were asked to judge their personal emotional state on a continuous linear scale ranking from 0 to 10 cm in two subscales: (1) pleasant: animated, inspired, enchanted, comfortable, happy, pleased, cheerful, and calm and; (2) unpleasant: uncomfortable, angry, sad, afflicted, frightened, tense, annoyed, and worried [53].

#### 2.2.3. Facial Emotional Recognition Task

This test has been used previously in other studies, both in schizophrenic patients and epileptic patients [36, 54]. Facial emotional expressions of 8 models (4 males and 4 females) from Ekman and Friesen [55] were presented on a computer screen (15 in). Each model portrayed six basic emotions (happiness, sadness, anger, fear, surprise, and disgust) and one emotionally neutral expression. Participants were seated at a distance of 60 cm from the computer screen. They were instructed to press a key and, at the same time, to label the emotion that each stimulus represented, performing it as precisely and quickly as possible. Presentation of each one of the 56 stimuli lasted until the subject responded with a maximum of 2000 ms. A list with the possible emotional expressions represented in the photographs was shown to them previously, and it was confirmed that they had understood the instructions completely. Also rehearsal essays were performed before the task. Response times were recorded on a computer at the moment the subjects pressed the key. The answers were written down by hand by the experimenter.

A facial identity recognition task was presented. The goal of this task was to control for possible difficulties in face configuration processing in schizophrenics that may affect emotional recognition. Participants were asked to press a key when a previously indicated person appeared (28%) on the computer screen, with no regard for the emotional expression. Presentation of each of the 56 stimuli lasted until the subject responded with a maximum of 2000 ms. number of correct responses and response times were recorded in the computer.

#### 2.2.4. Prosodic Emotional Recognition Task

This test consisted of 32 sentences in Spanish presented on a computer. The semantic content of the sentences was emotionally neutral but were spoken with different affective prosody—happy, sad, angry, and fear—by two professional radio announcers, one male and one female (e.g., “The table is square”; “The moon is a satellite”). The stimuli with different emotional intonation were presented randomly. Participants were asked to label the emotional tone expressed in the sentences, given a list with the four possible emotions. Answers were recorded by the experimenter to determine the number of correct responses. This task has been used in other experiments, and tested previously in a normal sample [36, 54].

#### 2.2.5. Social Context Emotional Recognition Task

Four films with an approximate duration of 2 min were presented. All in Spanish, without music; they included several characters, and the dialogues represented situations for each one of the four aforementioned emotions (happiness, sadness, anger and fear). The films had been used in a previous study [36] and were presented randomly on a computer screen (15 in). Each participant was seated at a distance of 60 cm while watching the stimuli. At the end of each film, participants were asked to respond to a questionnaire to determine their ability to describe the scene, identify the emotions expressed by the principal and secondary characters, and indicate their perception of the intensity with which the characters experienced that emotion. In addition, participants were asked to indicate their emotional reaction when watching each film and the intensity they experienced (empathy). To evaluate the emotional intensity of the characters and that experienced by participants, an emotional scale, similar to the Emotional States Scale mentioned above, was applied to the participants. It consisted of a continuous line 10 cm long, on which they had to make a mark; the lowest intensity corresponded to the extreme left (0 cm) and the greatest intensity to the extreme right (10 cm).

#### 2.2.6. Statistical Analysis

In order to measure sex differences in clinical symptomatology, one-way Analysis of Variance (ANOVA) test was applied for BPRS and PANSS. Two-way ANOVAs for independent groups were used in order to compare the data obtained from SCH and CON groups (Factor A) as a function of sex (Factor B) for: (a) sociodemographic data (b) Affective States Scale scores: for pleasant and unpleasant (c) identity and (d) social context emotional recognition: attribution of intensity to emotional expression of the principal character and empathy level experienced by the participant. A three-way ANOVA—A: groups; B: sexes; C: emotions—for mixed designs was applied to analyze the facial emotional recognition task: number of correct responses and response times and prosodic recognition task: number of correct responses. In order to assess the effect of age on facial and prosodic recognition, an Analysis of Covariance (ANCOVA) was applied, since females were older than males. Significant levels were adjusted using the Greenhouse-Geisser epsilon correction for factors, and corrected *P* values are reported. The percentage of congruent responses with that represented in the scene, identification with the principal character, and association of the scenes with their personal life were evaluated by means of a chi square test.

## 3. Results

### 3.1. Sociodemographic Data

Age was significantly different between sexes (*F*(1,72) = 23.10, *P* < .001). Schizophrenic females (SCHf) were about 8 years older than males (SCHm) and, as a result, the matched control females (CONf) compared to males (CONm). No differences were found in years of formal education (Table 1).

### 3.2. Clinical Characteristics

With regard to the initial psychopathological assessment, no differences either in BPRS or PANSS were found in the patient group. In the second assessment, SCHm showed higher scores than SCHf only on the Negative Symptoms Subscale (*F*(1,34) = 2.23, *P* < .03). (see Table 2).

 There were no differences between SCHf and SCHm in the number of previous hospitalizations, duration of illness, number of days between the initial and second evaluations, or in the type of medication. Two SCHf and two SCHm were medicated with 15 mg/day of haloperidol (250 chlorpromazine equivalent) while 18 SCHf and 16 SCHm were taking 10 mg/day of olanzapine (200 chlorpromazine equivalent).

### 3.3. Affective States Scale

**Figure 1:**
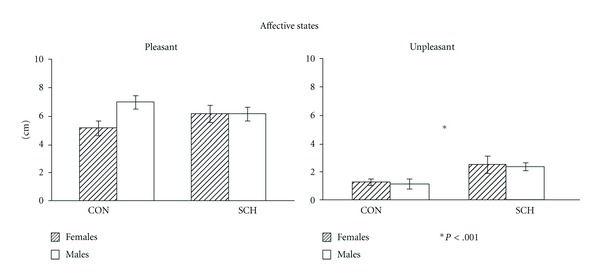
Means and standard errors on the pleasant and unpleasant Affective States Scale in controls (CON) and schizophrenic patients (SCH). SCH group reported higher unpleasant affectivity than CON.

There were no significant differences between SCH and CON, or between males and females, on the pleasant affective states subscale (*F*(1,72) = 0.06, *P* < .79); however, the SCH group reported higher unpleasant affectivity than CON (*F*(1,72) = 16.53, *P* < .001), both in the case of males and females at the beginning of the assessment session. No sex differences were observed. (Figure 1).

### 3.4. Emotional Recognition

#### 3.4.1. Facial Recognition

There were no significant differences on the identity control task between CON and SCH, or between sexes in terms of the number of correct responses and response times.

**Figure 2:**
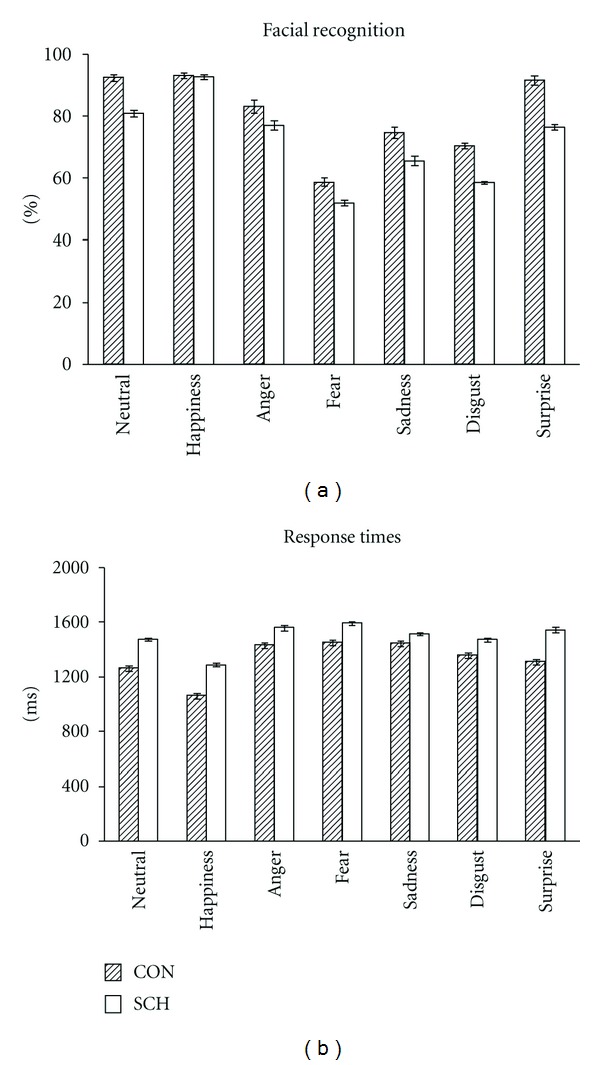
Means and standard errors of the percentage of correct responses and response times in the facial emotional recognition tasks for the control (CON) and schizophrenic groups (SCH).

Figure 2 shows that SCH achieved a lower number of correct responses for all emotions with the exception of happiness (*F*(1,72) = 13.74, *P* < .001) compared to CON as a main factor. With regard to sex, the statistical analysis did not reach significant values. Females in both groups CON and SCH, showed a tendency to have a higher number of correct responses in sadness recognition than males. The factor of emotions showed significant differences (*F*(6,432) = 36.92, *P* < .001) in the number of correct responses. As expected, happiness had the highest values and fear the lowest (Figure 2).

With respect to response times, SCH were slower to recognize all the emotions than CON (*F*(1,72) = 7.36, *P* < .008). There was no significant effect of sex. Response times were also significant between emotions (*F*(6,432) = 24.27, *P* < .001), with happiness showing the fastest response times (Figure 2). There were no significant interactions for either the number of correct responses or response times.

When age was considered as a covariate, the number of correct responses was still significant for emotions (*F*(6,432) = 3.1, *P* < .01) and for groups (*F*(1,72) = 12.81, *P* < .001). Although gender did not reach the level of significant differences, when age is considered as a covariate, it showed a trend (*F*(1,72) = 3.58, *P* < .06). There were no significant interactions between factors.

#### 3.4.2. Prosodic Recognition

**Figure 3:**
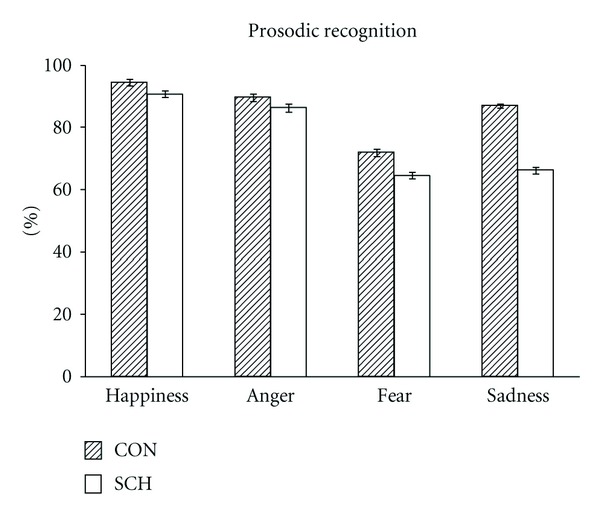
Means and standard errors for the percentage of correct responses in the prosodic emotional recognition task for males and females from the control (CON) and schizophrenic groups (SCH).

There were significant differences between emotions on the prosodic recognition task (*F*(3,1892) = 31.21, *P* < .001). SCH showed a significantly lower number of correct responses in prosodic emotional recognition than CON (*F*(1,72) = 16.53, *P* < .001) as a main factor. In addition, an interaction of emotion by group was found (*F*(3,1892) = 4.24, *P* < .01). No sex differences were observed. As can be seen in Figure 3, the easiest emotion to recognize was happiness, whereas the most difficult one was fear. SCH showed a lower number of correct responses than CON with respect to all emotions. In addition, results demonstrate that only for SCH was recognizing sadness as difficult as identifying fear.

The ANCOVA with age, included as a covariate, revealed a significant effect (*F*(1,71) = 9.05,  *P* < .004). The significant differences between emotions in prosodic recognition disappeared (*F*(1,71) = 2.21, *P* < .10). The main effect for group remained, with SCH showing a significantly lower number of correct responses in prosodic emotional recognition than CON (*F*(1,72) = 15.79, *P* < .001). Although no sex differences were observed, an interaction of group by gender was found (*F*(1,71) = 4.06, *P* < .04). A Pearson correlation analysis between the global number of correct responses and age revealed a significant negative correlation only for SCHm (*r* = −0.52, *P* < .005).

#### 3.4.3. Social Context Emotional Recognition

All the participants in CON and SCH groups were able to recognize all the emotions expressed by the characters in the 4 films.

**Figure 4:**
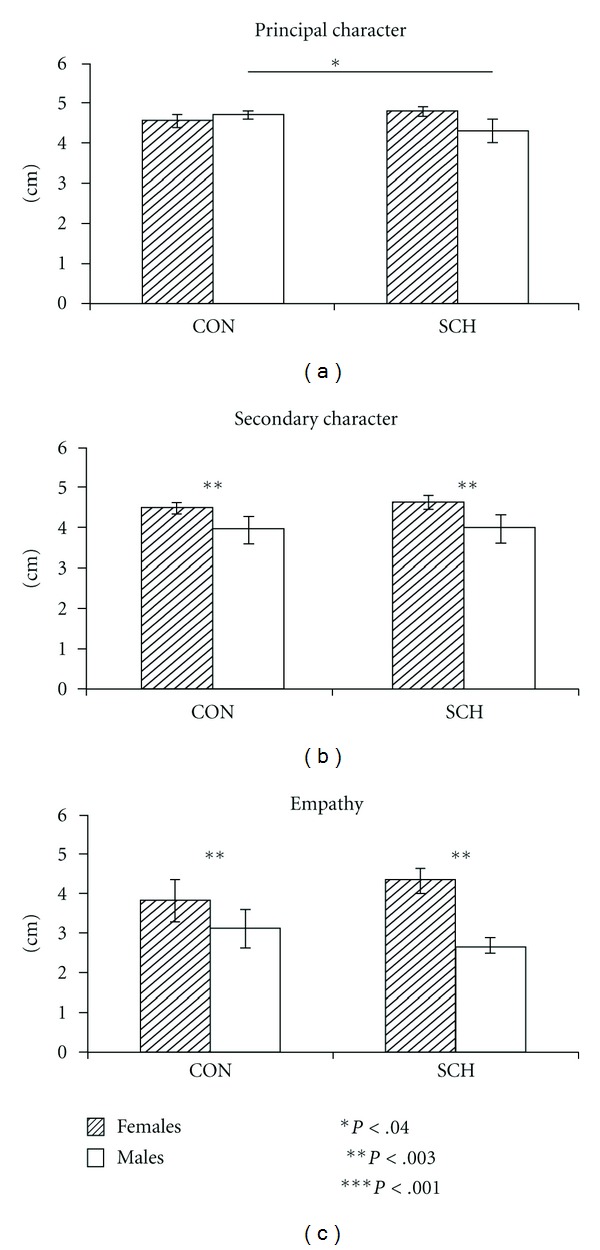
Means and standard errors for intensity level attributed to happiness expression from the principal and second characters and the empathy level experienced during the happiness film for females and males of the control (CON) and schizophrenic groups (SCH).

 With respect to the intensity of the emotions expressed by the principal character, there were no differences between CON and SCH or between the sexes, but a significant interaction of group by sex (*F*(1,72) = 4.18, *P* < .04) revealed that SCHm attributed less intensity to the expression of happiness by the principal character than CONm. Both CON and SCH females attributed a higher intensity to the expression of happiness by the secondary character (*F*(1,72) = 9.35, *P* < .003) and reported greater empathy with the characters in the films when they expressed happiness, compared to males in both groups (*F*(1,72) = 11.94, *P* < .001) (see Figure 4).

**Figure 5:**
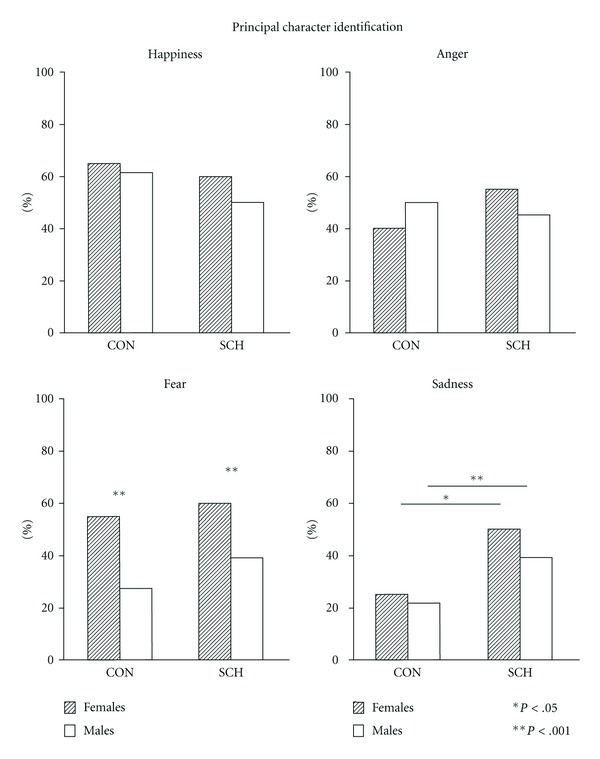
Percentage of controls (CON) and schizophrenic patients (SCH) that reported being identified with the principal character.

A higher percentage of females compared to males in both groups reported identifying with the principal character in the case of fear (*P* < .001); and a higher percentage of SCH females and males identified more closely with sadness (*P* < .05, *P* < .001, resp.), compared to CON. Similar results were found in fear and sadness in terms of the percentage of subjects when they relate the films scenes to their personal lives (*P* < .001 in all cases) (Figure 5).

## 4. Discussion

The purpose of the present study was to determine sex differences in schizophrenic patients with regards to emotional recognition, considering three modalities: facial, prosodic and in a socioemotional context, as well as to confirm impairments in those modalities in early onset schizophrenic patients.

### 4.1. Sociodemographic and Clinical Characteristics

As has been reported by Kraepelin in 1919, and other authors have replicated [2, 3, 56], both illness onset and first hospital admission occurred later in schizophrenic females than in males by approximately 8 years. More males than females were single or had no stable partner; half of the patients, both females and males had jobs; some males were students, while some females were homemakers. In general, patients showed adequate social functioning, perhaps due to the recent onset of the illness.

Regarding psychiatric symptoms, no differences were observed between the sexes when the patients arrived at the hospital in the acute phase of the illness. Although other studies have affirmed that males present more acute symptomatology, and a higher prevalence of paranoid symptoms, aggression and antisocial behaviour [7] compared to females, we did not find similar results.

However, in the second psychopathological assessment, during the remission stage, males showed higher scores of negative symptoms than females. The better outcomes for the latter have been associated with better responses to treatment, a higher number of spontaneous remissions and the later onset of the illness, compared to males [7, 13, 15]. Rao and Kölsch [57] have attributed the higher severity in negative symptoms in males to lower concentrations of estradiol.

Turning to emotional states, we observed no differences by sex in the Affective States Scale scores. While both groups showed higher scores on the pleasant scale in comparison to the unpleasant scale, schizophrenics reported a higher level of unpleasant states. This finding is significant, since a negative emotional state could work as a marker for overlooked negative emotions on neutral faces. For instance, studies in depressive patients have described that they tend to attribute sadness to faces showing neutral expressions [58].

In conclusion, sex differences in schizophrenic patients were observed only in negative symptoms in the remission stage after medication, with a higher level in males than females.

### 4.2. Facial Emotional Recognition

Schizophrenic patients showed lower accuracy than controls in the recognition of all emotions, with the exception of expressions of happiness. The main effect for group remained when age was introduced as a covariate, indicating an independence of emotional recognition abilities in relation to age. In addition, the schizophrenic patients presented longer response times. These findings agree with those reported by several other studies in which deficits in facial emotional recognition in schizophrenic were found [38, 39]. It has been documented that people with schizophrenia commit more errors in recognizing fear, disgust and sadness, though they have no problem in recognizing happiness, an emotion that is more easily recognized than others [26, 59]. The fact that there were no difficulties in identity recognition suggests that this is not a problem in facial configuration processing.

Another issue has to do with response time. It is commonly accepted that schizophrenic patients generally show a slower processing on most type of tasks, including facial emotional recognition. In previous studies, we have found slower reaction times in male schizophrenics than healthy controls in odd-ball tasks using letters and emotional faces as stimuli [60], as well as on identity and emotional tasks [61]. In addition, longer reaction times (RT) were correlated with higher level of symptomatology and lower number of correct responses on both tasks [60].

On the question of differences between the sexes in emotional recognition, the variation found did not reach significant values. However, when age was introduced as a covariate, a trend was observed in the sense of a better performance in females than males. Other studies have reported better facial emotional recognition in females than males (i.e., [26, 62, 63]). In particular, it has been reported that healthy females outperform males in recognizing sad faces, while males perform better when it comes to identifying fear [31].

### 4.3. Emotional Prosody

In this research, schizophrenics showed lower accuracy in prosodic emotional recognition than controls as a whole. These results are congruent with those of other studies [34] that also found significant deficits in sadness and fear prosodic recognition in schizophrenics.

In a previous study, we have also found difficulties in prosodic recognition of happiness and sadness in chronic resistant schizophrenics without treatment [36]. However, a significant improvement was observed in the patients after they took olanzapine, as was a reduction in depression, though only for happiness prosodic expressions [36]. The effect of medication is relevant, since almost all the schizophrenics in the present study had taken olanzapine for at least 15 days when they were evaluated; therefore, accuracy in identifying happiness may have improved.

Regarding sex differences in prosodic recognition, no differences were found as long as age was not considered as a covariate; however, when it was, a significant group by gender interaction was found. In addition, a negative correlation between accuracy and age was observed only for schizophrenic males. These results suggest that prosodic recognition depends on age, mainly in schizophrenic males, as their performance worsens as age increases.

The prosodic stimuli used in this work had the particularity that the sentences were semantically neutral, such that the participants only had to pay attention to the emotional prosody. It is relevant because Schirmer et al. [32, 64] proposed that sex differences in emotional prosody depend on whether the subjects are asked to pay attention only to prosody and not to semantic information in the sentence. When the subjects pay attention to semantic content and emotional intonation simultaneously, females can integrate the two types of information more quickly than males, but when they only have to attend to emotional prosody, there are not sex differences. In daily life, speech contains both types of information that must be processed simultaneously, so it would be useful to evaluate emotional prosody processing when semantic information is also present, or even when there is incongruence between them.

Disorders in prosodic emotional recognition are related to difficulties in maintaining social interaction, which leads to social withdrawal even in healthy young adults [65]. In this sense, these disorders would result in lower social adaptive behavior in schizophrenic males than females as age increases.

### 4.4. Emotional Recognition in a Social Context

Understanding information on socioemotional contexts requires several abilities, including, as a first step, recognizing facial and body expressions and emotional prosody, and second, integrating those different types of emotional information. In addition, it requires interpreting all the emotional information within a context that includes, on the one hand, physical and spatial features, but more importantly, the ability to infer mental states and intentions from other individuals, in order to predict their behaviour and interpret the significance of personal relationships, as well as the effect of one's own behavioral responses on others. The final step refers to the empathetic abilities needed to understand the feelings of others (for reviews [66, 67]).

With regard to the recognition of the emotion represented in the films, all participants were capable of reporting their content congruently. These may be due to the fact that there are several simultaneous sensory inputs that allow the patients to compensate possible difficulties in any particular input channel. However, schizophrenic males attributed less intensity to the expression of happiness by the principal character than control males. In addition, both CON and SCH females attributed higher intensity to the expression of happiness by the secondary character. A higher percentage of females compared to males in both groups reported identifying with the principal character in the case of fear, while schizophrenic females and males identified more strongly with sadness than did controls. Similar results were found in the percentage of subjects when they were asked to relate the films scenes with their personal lives. Furthermore, both control and schizophrenic females reported a greater intensity of empathetic feelings during the happiness film. These results are consistent with those of a previous study carried out by Grossman and Wood [68] in which females reported experiencing emotions with greater intensity than males in findings that correlated with higher electromyograph activity.

Both female and male patients showed higher identification with the principal character in sadness films and reported that this scene related more closely to their personal lives than did controls. This may be related to the more unpleasant emotional state reported by the patients on the Affective Scale.

In a previous study conducted with similar facial, prosodic, and social context stimuli, we found that schizophrenic people showed difficulties in making social judgments as to what was happening in the scene, and in attributing intentions to the characters portrayed [36].

The role of emotions in formation and maintenance of interpersonal relationships is crucial. Not surprisingly, then, disturbances in an individual's emotions have important social consequences [69]. Understanding facial expressions in others makes it possible to form a representation of their intentions and emotional states and then respond accordingly in an adaptive way. Although there exists an important relationship between emotional recognition and adequate social development [69], it is also suggested that other cognitive abilities, such as executive functions, are fundamental to interpreting emotions in a scene where the role of lateral prefrontal cortex is predominant [70, 71].

It is important to conduct studies with complex emotions such as proud, shame, guilt, frustration, and so forth, because, in daily life, patients are continuously exposed to not only basic emotions, but also complex ones. It is possible that when exposed to such complex emotional expressions schizophrenics would show greater impairment and more pronounced differences between sexes.

This study took into account the fact that there are many variables that can influence emotional recognition performance; however, controlling for all such variables made it difficult to recruit a larger sample. For this reason, there is some question as to what degree these findings could be generalized to a larger population.

In summary, in the results of the present study, both females and males schizophrenic patients showed poorer performance than controls in terms of visual and prosodic modalities with respect to almost all emotions, except for happiness. However, when placed in the social context portrayed in the films, patients with schizophrenia were able to recognize all the emotions, suggesting that simultaneous sensory inputs may allow them to compensate for the difficulties that can appear when information is processed by independent input channels. Schizophrenic patients reported that they identified more with sad films than did controls, a finding that may be related to a more unpleasant emotional state. Schizophrenic females and males presented similar impairments with regards to facial and prosodic emotional recognition compared to CON, though upon considering age as a covariate, schizophrenic males showed poorer performance as age increased. However, in the social context of emotions, females showed higher empathy in relation to happiness than males in both groups and attributed a greater intensity of happiness to the secondary characters. As well, control and schizophrenic females identified more with fear than males.

Knowledge of emotional impairments in schizophrenia may be useful in designing therapeutic strategies, by taking into account the emotions that present greater difficulties in a particular sensory modality. It would be important to attend not only to emotional recognition of faces and prosody impairments, but to the way the patients interpret those emotions in order to improve formation and maintenance of interpersonal relationships. Together with other findings, the data from this study suggest that both in clinical management and research most take into account sexual differences in schizophrenic patients.

## Figures and Tables

**Table 1:** Sociodemographic characteristics from healthy controls (CON) and schizophrenic patients (SCH).

	CON				SCH			
	Females (*n* = 20)		Males (*n* = 18)		Females (*n* = 20)		Males (*n* = 18)	
	$\overset{®}{\text{X}}$	SD	$\overset{®}{\text{X}}$	SD	$\overset{®}{\text{X}}$	SD	$\overset{®}{\text{X}}$	SD
Age (years)	34.20	±9.46	26.06	±7.16	36.15	±7.01	27.06	±7.22
Formal education (years)	13.87	±2.62	13.88	±2.83	13.87	±2.87	12.36	±2.58

% Subjects								

Single	30%		55%		35%		61%	
Married or stable partner	60%		33%		40%		33%	
Widow	0%		0%		5%		0%	
Divorced/separated	10%		11%		20%		6%	

Occupation	% Subjects							

Student	25%		33%		0%		22%	
Employee	5%		50%		45%		50%	
Professional	10%		17%		15%		6%	
House worker	55%		0%		30%		0%	
None	0%		0%		5%		22%	
Others (commerce)	5%		0%		5%		0%	

**Table 2:** Clinical characteristics of the schizophrenic patients; BPRS and PANSS were applied twice, first in the acute phase (1) and later in the remission phase (2).

	Females (*n* = 20)		Males (*n* = 18)	
	$\overset{®}{\text{X}}$	DS	$\overset{®}{\text{X}}$	DS
Number of Hospitalizations	1.65	±1.18	1.44	±.61
Duration of illness (months)	22.50	±16.87	24.44	±13.57
Days of Hospitalization at the time of evaluation	14.25	±4.12	15.33	±5.58
BPRS (1)	44.89	±16.46	45.94	±7.32
PANSS positive (1)	24.00	±7.10	25.66	±5.96
PANSS negative (1)	21.52	±7.16	23.33	±7.06
PANSS general (1)	36.16	±9.48	37.16	±6.4
PANSS global (1)	81.75	±19.40	86.16	±10.6
BPRS (2)	24.52	±7.61	25.29	±8.04
PANSS positive (2)	8.36	±4.25	9.56	±2.92
PANSS negative (2)*	11.47	±5.18	14.93	±3.67
PANSS general (2)	20.42	±8.96	22.00	±2.36
PANSS global (2)	44.73	±9.56	47.35	±7.94

*P < .03.
